# How to Make Primary Healthcare More Popular: Evidence from the Middle-Aged and Elderly in China

**DOI:** 10.3390/healthcare10091783

**Published:** 2022-09-16

**Authors:** Liping Fu, Ya’nan Fang, Shu Yang, Yanqing Xu

**Affiliations:** 1 College of Management and Economics, Tianjin University, Tianjin 300072, China; 2 Center for Social Science Survey and Data, Tianjin University, Tianjin 300072, China; 3 College of Politics and Public Administration, Qinghai Minzu University, Xining 810007, China; 4 School of Public Administration, Hainan University, Haikou 570208, China

**Keywords:** primary healthcare, community, combined medical care and pension services in community, middle-aged and elderly adults, structural equation model

## Abstract

Since 2001, China has been an aging society; it is expected to become superaged by 2033. This rapid aging trend poses a challenge to the elderly regarding their pension services and healthcare. Primary healthcare has great potential for serving older adults in the community, yet it is not popular. This study used 1977 samples from the 2018 China Health and Retirement Longitudinal Study database to explore the use of outpatient services in primary care institutions among the middle-aged and elderly. Using a structural equations model, we constructed a framework to explore pathways leading to primary outpatient use. We discovered that the supply of primary health services had a significant direct and mediating effect on the utilization of primary outpatient services, and that community pension services may indirectly discourage it. In addition, the supply of primary health services has a suppressor effect between medical insurance and primary outpatient utilization. Health insurance directly promotes primary outpatient utilization, while the supply of primary care institutions suppresses the positive influence of medical insurance on the utilization of primary outpatient services. Therefore, community pension services should pay attention to differentiated services. Moreover, adjusting the coordinated development of medical insurance and the supply of primary healthcare could enhance the positive effects of medical insurance for outpatients.

## 1. Introduction

The vital role of primary care in achieving the goal of healthcare for all is identified in the Alma-Ata Declaration [1]. With the launch of the New Health Care Reform (NHCR) in 2009, the Chinese government emphasized strengthening urban and rural primary healthcare (PHC) and providing basic public health services [2]. The “Healthy China 2030 Plan Outline” also emphasizes the development of PHC. The shift to a focus on PHC—village clinics, town health centers, and urban community health centers—is expected to relieve the pressure on the healthcare system by providing inpatient care and basic services at the local level [3]. Nevertheless, PHC is not very popular. In 2020, outpatients at primary care institutions (PCIs) accounted for 23.9% of all outpatients. The number of inpatients at PCIs accounted for 16.1% of the total [4]. Because more people tend to go to high-level hospitals for treatment [5], the existing medical system suffers from the “Matthew Effect”: high-level hospitals become stronger, while PCIs have little room to grow [6]. Furthermore, guiding patients to PHC can help to achieve universal health care (UHC) [7] and reduce readmissions [8]. Therefore, studying PHC use has practical significance.

Studies so far have focused mainly on two aspects of PHC: optimization and use. Optimization was mainly discussed regarding primary care physicians [9], services for the most vulnerable [10,11], and the number of institutions and beds [12], all of which have implications for PHC [13,14,15]. In addition, studies have also identified health insurance [16], chronic disease [17], policy formulation [18], and medical alliances [19] as significant influencing factors. In China, PHC is expected to play an initial diagnostic role to avoid over- and under-use of healthcare. However, the negative consequences of combined community medical care and pension services have rarely been discussed. In fact, medical services are offered in the same building as pension services. Combined medical care and pension services, which the government proposed in 2014, are designed to improve service at geriatric nursing institutions. Because they often operate near the community, there is some overlap between combined medical care and pension services and PHC. On the other hand, most studies focus on the direct effect of medical resources on PHC use, but they do not consider that medical resources are also affected by economic policies or other services.

Our study explored two questions: (1) What role does the combination of community medical care and pension services play in the use of PHC? (2) Does PHC supply play a mediating role? 

## 2. Research Framework 

**Figure 1:**
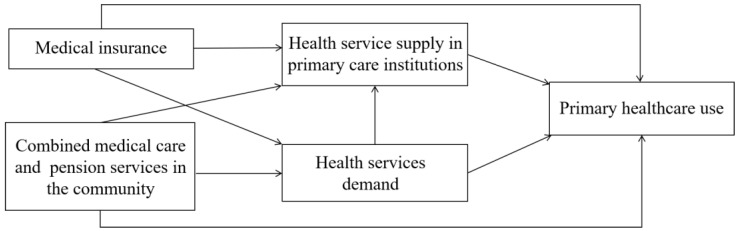
Analysis framework. The amount of healthcare services in PCIs should be read as the status of medical resources in the area where the individual samples are located. Combined medical care and pension services in the community are also read in this way.

From studies on the influencing factors of PHC use, we identified multiple factors. The analysis framework is shown in Figure 1.

### 2.1. Medical Insurance

The public’s use of healthcare services is determined to a large degree by insurance [20,21]. The reimbursement rate of PCIs in many provinces is higher than that of tertiary medical institutions, so patients may be inclined to choose to go to a PCI [22], which indicates that there may be a positive relationship between medical insurance and PHC. At the same time, the price mechanism of medical insurance can aid the allocation of medical resources [23]. Health departments usually use different insurance policies to allocate resources. Matsushima pointed out that with the expansion of medical insurance coverage, the supply side needs to be prepared for increased demand [24]. China’s medical insurance coverage rate has reached more than 95%, but it is still difficult for people to see a doctor. As primary medical insurance becomes more popular and widespread, the supply of primary health services will need to be adjusted accordingly. Zhou, Guo, and Bai pointed out that national and private medical insurance has a positive effect on people’s health [25,26,27,28], and our study used a patient’s health status to represent service demand. 

### 2.2. Combined Medical Care and Pension Services in the Community

For the middle-aged and elderly, healthcare is provided by combined community medical care and pension services [29], which affects the use of PHC services; however, to a certain extent, it also alleviates the difficulty in seeing a doctor [30]. Moreover, in the case of insufficient medical resources, the health department may consider existing services and an individual’s needs when allocating resources to a PCI. Thus, we inferred that when the combined services are better, the supply of PCIs could decrease. Ding’s research shows that combined services could meet the diverse needs of the elderly [31]. 

### 2.3. Healthcare Services in Primary Care Institutions 

Healthcare services in PCIs are an objective means by which to measure the PHC system and the factors influencing patients’ healthcare decisions [13,20]. Some studies found a tendency to reduce willingness to use PHC due to poor service quality in PCIs [32,33]. 

### 2.4. Healthcare Service Demand 

To meet the population’s needs and increase the use of PHC, the health department has been working hard to improve supply [34]. As the expected use of PHC changes with the aging process [35], some countries need to improve chronic disease management [36,37]. Because of increasing demand, not all patients are able to go to a PCI [38], so they often have to select secondary or tertiary hospitals [39]. 

## 3. Materials and Methods

### 3.1. Data

The China Health and Retirement Longitudinal Study (CHARLS) [40] and the China Health Statistics Yearbook were combined to study PHC use among the middle-aged and elderly. CHARLS collected high-quality microdata organized by Peking University that represented families and individuals aged 45 and over. The survey used the Probability Proportionate to Size (PPS) sampling method covering 150 counties containing 450 villages that had 17,000 people in about 10,000 households. Investigators conducted the survey in four waves––2011, 2013, 2015, and 2018––with 2011 serving as the baseline [41]. Data from the 2018 questionnaire were released in 2020. The response rate for respondents aged 60 and over was 99%. The 2018 data reflected recent behavior, which was more meaningful for policy making. The questionnaire included basic personal information: family structure, financial support, health status, physical measurement, healthcare use, medical insurance, work, retirement and pensions, income, consumption, and community conditions. From the CHARLS database, this study extracted information on the state of community services (combined medical care and pension services), the individual health status (health service demand), and healthcare use (medical insurance and the use of PHC). At the same time, medical resource supply data of the region were extracted from the Health Statistics Yearbook. As PHC mainly provides outpatient care, samples that did not include outpatients were excluded. In the end, 1977 samples were retained.

### 3.2. Measures

According to the research framework, the latent variables were divided into five categories: medical insurance, combined medical care and pension services in the community, supply of PCIs, the use of PHC, and health service demand. These were then set for 11 observation variables. The latent variable is represented by λi, and the observed variable is represented by ρij (Table 1). Medical insurance in PHC (λ1) [42], as the main form of compensation for the economic nature of medical resource utilization [43], had a significant impact on individual medical behavior [44,45]. It included the reimbursement rate of medical insurance (ρ11) and the type (ρ12). The reimbursement rate is the percentage of expenses paid by the Medical Insurance Bureau over total medical costs. The descriptive statistics (Table 1) indicate a large gap in economic compensation for PCIs. Combined medical care and pension services in the community provided the elderly with “close to home, elderly care combined with medical care” [46] and relieved the difficulty in seeking medical care [47], which in turn affected the behavior of seeking medical care. Therefore, this study selected medical services in the community pension center (ρ21) and the use of pension care in the community (ρ22) as observed variables. Statistics showed that relatively few elderly adults use pension services in the community, and the number and quality of PCIs are crucial factors affecting the choice of institutions [20,48,49]. Among them, the number of PCIs (ρ31), the number of beds (ρ32), and the number of personnel (ρ33) characterized the capabilities of PCIs. Physical characteristics are fundamental factors that promote the use of medical resources [50,51], while chronic diseases and activities of daily living (ADL) are often proxy variables [52,53]. For hypertension, patients tend to choose PCIs [54]. According to survey data from the Chinese Center for Disease Control and Prevention, two-thirds of the elderly suffer from chronic diseases. Therefore, the number of chronic diseases (ρ41) characterized the physical status of the elderly [55,56] and their health service demands. ADL and IADL (instrumental ADL) reflected the basic physical conditions of the middle-aged and elderly, including basic physical activities and key independent-living skills. Table 1 shows that the proportion of PHC used by middle-aged and older adults is low, at 30.7%.

### 3.3. Statistical Analysis

Data analysis was performed using SPSS ver. 22.0 (IBM Corp, Armonk, NY, USA) and AMOS ver. 24.0 (IBM, Armonk, NY, USA). We used KMO and Bartlett to measure whether the 11 observed variables were suitable for structural equation modeling. The test results are shown in Table 2. Kaiser’s research concluded that if KMO is less than 0.5, it is less suitable for factor analysis [57]. The Kaiser–Meyer–Olkin (KMO) value was 0.640 > 0.5, which meant it was suitable for factor analysis. The significance of Bartlett’s sphere test was *p* = 0.000 < 0.001, and the significance was at a high level. The data were suitable for the structural equation model.

The goodness-of-fit was described from three aspects, as shown in Table 3: absolute fit, value-added fit, and simple fit. For the absolute fit, the study selected five related indicators to measure: the root mean square residual (RMR), the root mean square of the approximate error (RMSRA), the goodness-of-fit index (GFI), the adjusted goodness-of-fit index (AGFI), and Χ^2^/df. These indicators conformed to the reference standard, indicating that the structural equation model was acceptable [58]. Regarding the value-added fit, the study selected five indicators—the normed-fit index (NFI), relative fit index (RFI), incremental fit index (IFI), Tucker–Lewis index (TLI), and comparative fit index (CFI)—to test the value-added fit of the model. The results showed that the five indicators all conformed to the reference standard. For the degree of simplicity, this study used two thrift adjustment indexes as measures that also conformed to the reference standard. In summary, this was an acceptable model.

## 4. Results

**Figure 2:**
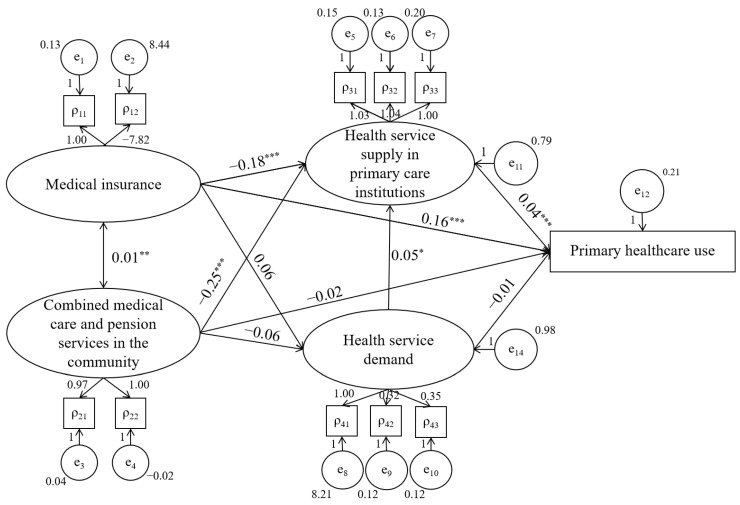
Structural equation model (Note: * *p* < 0.1; ** *p* < 0.05; *** *p* < 0.01).

The final fit-corrected model (Figure 2) shows the interrelation of the five variables, valid path, and effect values (Table 4). It concludes with 10 direct paths and seven indirect paths, of which one path passed the 0.1 significance; four paths passed the 0.05 significance; and six paths passed the 0.01 significance test.

### 4.1. Direct Path

First, medical insurance significantly affects the supply of PCIs and the use of PHC. It is worth noting that medical insurance harms supply but positively affects use. This means that the better the benefits of primary medical insurance, the lower the supply of PCIs. 

Second, the community’s combined medical and pension services negatively affect the supply of PCIs, thereby showing that the two types of service have a substitution effect. When the supply of PCIs is insufficient, the combined medical and elderly care services are critical and vice versa. The medical nature of the two services is an important reason for the substitution effect on the elderly. 

Third, health service demand positively affects PCI health services. Among them, chronic diseases are the most significant. The effect of health service demand on PHC use is not significant. 

Fourth, the supply of PCIs has a significantly positive effect on PHC use, which is guided by the quality and quantity of resources. The supply of PCIs is more than that of secondary and tertiary hospitals, which reduces the transportation cost for most patients. Moreover, if there are more primary health technicians, the quality of services in PCIs could be increased (service diversity and ability improvement brought by a personnel competition mechanism). The better the PHC, the more likely patients are to use PHC.

### 4.2. Indirect Path

Among the seven indirect paths, bootstrap was used to test the significance of the mediation effect. We found five significant indirect paths, and the intermediary variable is the supply of PCIs. In detail, medical insurance and combined medical care and pension services harm the indirect path to PHC because of the negative effects of medical insurance and combined medical care and pension services on supply in PCI. However, this path does not indicate that promoting PHC use by reducing the strength of medical insurance is optimal. The result only reflects the reality of 2018. The elderly who use combined medical care and pension services reduce their use of PHC because there is a substitution effect between the two services. Moreover, healthcare demand promotes health service supply, thereby promoting PHC use. When their needs are met, patients will use PHC.

## 5. Discussion

PHC aims to improve the equality and accessibility of healthcare [19]. Especially during the pandemic, PCIs were essential for primary diagnosis and referral [59,60]. However, PHC is often unpopular. The contributions of our study are mainly in two aspects. First, unlike previous studies on the influencing factors of PHC visits, we explored their indirect and direct effects, which clarified the mixed impact of current measures to promote primary care at PCIs. It included PCI infrastructure [61], financial compensation [62], combined community medical care and pension services, and personal health characteristics [63]. In particular, it supplements research into the role of combined services. Second, research into the reasons for using PHC will be essential for realizing equitable use. For other countries with underdeveloped PHC, considering the effect of mixed factors may guide health departments to formulate policies and plans to popularize the use of PHC. This study shows that the supply of health services is a significant intermediary variable. Medical insurance and combined medical care and pension services are also significant. 

### 5.1. Medical Insurance in PHC and the Use of PHC

The direct and indirect effects of medical insurance on PHC use have opposite results. First, it has: a direct, positive effect, which shows that economic support has a positive effect. YJ’s research confirms this [64]. However, when the PCI supply is used as a mediating variable, the indirect effect is negative. This phenomenon is called “the suppressor effect”. The main factor is that medical insurance limits the supply of PCIs because high-level hospitals siphon off resources [65]: the higher the medical insurance, the more that patients will choose secondary or tertiary hospitals, which leads to a shortage of primary medical resources.

At the same time, we discovered that this may also be related to regional outpatient reimbursement policies, which are determined by a threshold fee. Some regions often use medical insurance policies to guide patients to use PHC, which lessens the demand for high-level hospitals to develop PHC. For example, the primary medical resources of Henan province, a region with a medium level of medical care, are worse than those of Beijing, but the outpatient reimbursement rate in Henan province is 60%. In Beijing, 70% (non-PCIs) and 90% (PCIs) of outpatient expenses over RMB 1800 are reimbursed. If outpatient expenses do not reach a certain amount, the reimbursement rate in Henan may be higher. Therefore, medical insurance is an essential means for local health departments to guide the development of PHC. In addition, the amount of PCI health services significantly benefits PHC use, but the absolute number of financial subsidies for PCIs is smaller than for high-level hospitals [66]. With the increase in medical insurance coverage, the disadvantage of inadequately developed PHC becomes more obvious. Thus, the siphoning effect of high-level hospitals on medical resources and the insufficient development of PHC have opposite results on the direct and indirect effects of medical insurance on PHC use.

### 5.2. Combined Medical Care and Pension Services and the Use of PHC

LL pointed out that combined medical care and pension services solve the unmet medical needs of the elderly and the low use rate of PHC [67], which supports our findings. However, continuous improvement in the quality of PHC means that combined services no longer provide advantages for the elderly with self-care abilities. After PHC is fully used, they could provide different services, such as combined smart pension and medical care, to improve elderly health management [46]. If this were implemented, combined services and PHC could grow together. 

### 5.3. Limitations and Directions for Future Research

This study had some limitations. We only considered some of the influencing factors and paths for the use of PHC. Other influencing factors are income, consumption habits, medical behavior habits, and distance from medical institutions. However, the significance of our findings is not affected. Another possible limitation is that our sample of people who used PHC consisted of those who went to medical institutions but ignored those who did not. Future studies may use questionnaires for this and ask about the use frequency of outpatient services.

## 6. Conclusions

In this study, we confirmed that the supply of PCI health services has a significant mediating effect on PHC use. In addition, in this study, we found that, although combined medical care and pension services did not directly affect PHC use, they affected PHC use through the supply of PCI health services. Moreover, we found a substitution effect between combined services and PHC. To improve the current situation, combined services should instead provide intelligent health monitoring services, preventive care, and long-term care. Medical insurance positively influenced the use of PHC, but given its insufficient development, the insurance suppressed the supply of primary health services, which suppressed the positive influence of medical insurance on the use of primary outpatient services. To promote its use and make PCH more popular, medical insurance and the number of primary health services need coordinated development, and PCIs should be strengthened to manage chronic diseases. Taking full advantage of the positive effects of various factors could make PHC more popular in China.

## Figures and Tables

**Table 1:** Variable descriptive statistics.

Latent Variable λi	Observed Variable ρij	Definition	M (SD)/%
Medical insurance	Reimbursement rate	Medical insurance reimbursement expenses/Total cost (0–100)	20.202 (33.171)
	Type of reimbursement	1 = No insurance	62.67%
		2 = Urban employee medical insurance	6.98%
		3 = Urban and rural resident medical insurance	28.53%
		4 = Government medical insurance	1.01%
		5 = Private medical insurance	0.81%
Combined medical care and pension services in the community	Medical services in the community pension center	0 = No	95.5%
		1 = Yes	4.5%
	Use of pension care in the community	0 = No	95.5%
		1 = Yes	4.5%
Health service supply in primary care institutions	Number of primary care institutions	Number of primary care institutions per 10,000 people	7.186 (2.207)
	Number of beds in primary care institutions	Number of beds in primary medical institutions per 10,000 people	12.044 (3.514)
	Number of the physician in primary care institutions	Health technicians in primary care institutions per 10,000 people	28.442 (3.273)
Health service demand	Number of chronic diseases	Discrete variable	4.407 (3.032)
	ADL	0 = Without difficulty	67.78%
		1 = With difficulty	32.22%
	IADL	0 = Without difficulty	62.01%
		1 = With difficulty	37.99%
Outpatient use of primary care institutions during the past month		0 = No	69.3%
		1 = Yes	30.7%

**Table 2:** KMO and Bartlett sphericity test.

**KMO**		0.640
**Bartlett sphericity test**	X^2^	11,466.259
	df	55
	*p*	0.000

**Table 3:** Structural equation fit index.

Index	Fitting Index	Value	Standard
Absolute fit	RMR	0.061	<0.08
	RMSRA	0.031	<0.05
	GFI	0.994	>0.90
	AGFI	0.982	>0.90
	Χ^2^/df	2.941	1~3
Value-added fit	NFI	0.991	>0.90
	RFI	0.986	>0.90
	IFI	0.994	>0.90
	TLI	0.991	>0.90
	CFI	0.994	>0.90
Simple fit	PCFI	0.633	>0.50
	PNFI	0.631	>0.50

**Table 4:** Indirect path load factors.

Indirect Paths	Estimate	Bias-Corrected CI (95%)	*p*-Value
Medical insurance → Supply of PCIs → PHC use	−0.010	(−0.021, −0.004)	0.004 ***
Medical insurance → Health service demand → PHC use	0.000	(−0.005, 0.001)	0.423
Medical insurance → Health service demand → Supply of PCIs →PHC use	−0.009	(−0.019, −0.002)	0.011 **
Combined medical care and pension services in the community → Supply of PCIs → PHC use	−0.010	(−0.021, −0.004)	0.004 ***
Combined medical care and pension services in the community → Health service demand → PHC use	0.000	(−0.001, 0.007)	0.432
Combined medical care and pension services in community → Health service demand → Supply of PCIs → PHC use	−0.008	(−0.019, −0.002)	0.015 **
Health service demand → Supply of PCIs → PHC use	0.002	(0.000, 0.006)	0.025 **

Note: ** *p* < 0.05; *** *p* < 0.01.

## Data Availability

The datasets generated and analyzed during the current study are available in the CHARLS repository (http://charls.pku.edu.cn/en, accessed on 11 September 2022).

## Abbreviations

CHARLS: China Health and Retirement Longitudinal Study; SEM: structural equation model; ADL: basic or physical; IADL: instrumental basic or physical; KMO: Kaiser–Meyer–Olkin; RMR: residual root mean square; RMSRA: root mean square of the approximate error; GFI: goodness-of-fit index; AGFI: adjusted goodness-of-fit index; NFI: standard fitting index; RFI: relative fit index; IFI: incremental fit index; TLI: Tucker–Lewis index; CFI: comparative fit index; PCIs: primary care institutions; PHC: primary healthcare.
